# Correction to 24 papers to add an additional interest disclosure

**DOI:** 10.1093/ntr/ntae234

**Published:** 2024-10-15

This is a correction to the following papers:

- https://doi.org/10.1093/ntr/ntu144
- https://doi.org/10.1093/ntr/ntw388
- https://doi.org/10.1093/ntr/ntv014
- https://doi.org/10.1093/ntr/ntv104
- https://doi.org/10.1093/ntr/ntv237
- https://doi.org/10.1093/ntr/ntv235
- https://doi.org/10.1093/ntr/ntw158
- https://doi.org/10.1093/ntr/ntw236
- https://doi.org/10.1093/ntr/ntw282
- https://doi.org/10.1093/ntr/ntx017
- https://doi.org/10.1093/ntr/ntx073
- https://doi.org/10.1093/ntr/ntx102
- https://doi.org/10.1093/ntr/ntx145
- https://doi.org/10.1093/ntr/ntx285
- https://doi.org/10.1093/ntr/nty137
- https://doi.org/10.1093/ntr/ntz030
- https://doi.org/10.1093/ntr/ntaa010
- https://doi.org/10.1093/ntr/ntaa086
- https://doi.org/10.1093/ntr/ntaa102
- https://doi.org/10.1093/ntr/ntaa182
- https://doi.org/10.1093/ntr/ntaa224
- https://doi.org/10.1093/ntr/ntaa249
- https://doi.org/10.1093/ntr/ntad072
- https://doi.org/10.1093/ntr/ntad107

Between mid-2015 and 2020, Ray Niaura and David Abrams frequently communicated with Juul Labs personnel, for which they received hospitality in the form of meals at some meetings but no compensation. The authors apologize for not previously disclosing this hospitality to the journal or readers.

